# Unraveling the pathway from treatment burden to holistic health status in older adults with complex multimorbidity: the mediating roles of stress adaptation and depressive symptoms

**DOI:** 10.3389/fpubh.2026.1824738

**Published:** 2026-08-20

**Authors:** Yajun Shi, Jie Su, Yujia Fu, Ying Zhang, Yu Sheng, Huafang Zhang, Jing Shao

**Affiliations:** 1 Department of Nursing, The Fourth Affiliated Hospital of School of Medicine, and International School of Medicine, International Institutes of Medicine, Zhejiang University, Yiwu, China; 2 Institute of Nursing Research, Zhejiang University School of Medicine, Hangzhou, Zhejiang, China

**Keywords:** complex multimorbidity, depression, holistic health status, stress adaptation capacity, treatment burden

## Abstract

**Background:**

Patients with complex multimorbidity are prone to fall into the cycle of “burden-management failure-deterioration” under the overspending of resources due to the complex management burden of cross-system chronic diseases superimposed on frequent visits and polypharmacy.

**Aim:**

To investigate the relationship between treatment burden and holistic health status in older adults with complex multimorbidity, and to analyze the mediating effects of depressive symptoms and stress adaptation capacity on holistic health status.

**Methods:**

Participants were recruited from nine healthcare facilities across three cities in Zhejiang Province, China. Data were collected with a sociodemographic questionnaire, the Treatment Burden Questionnaire, Stress Adaptation Scale, 9-item Patient Health Questionnaire, and Long-Term Conditions Questionnaire. Parallel mediating effect modeling was performed using the Lavaan package in R software, and the mediating effect between depressive symptoms and stress adaptation capacity was tested using the Bootstrap resampling method.

**Results:**

A total of 507 participants with complete data were included in the mediation analysis. Median (IQR) scores for treatment burden, holistic health status, depressive symptoms, and stress adaptation capacity were 25.0 (12.0–42.0), 78.0 (66.0–88.0), 3.0 (1.0–6.0), and 20.0 (18.0–25.0), respectively. Treatment burden had a significant direct negative effect on holistic health status (*B* = −0.263, 95% CI [−0.357, −0.168]), with a total effect of −0.449 (95% CI [−0.538, −0.360]). Further mediation analysis revealed that depressive symptoms (*B* = −0.095, 95% CI [−0.145, −0.047]) and stress adaptation capacity (*B* = −0.091, 95% CI [−0.138, −0.051]) partially mediated this relationship.

**Conclusion:**

Stress adaptation and depressive symptoms partially mediated the relationship between treatment burden and holistic health status. Healthcare professionals can improve the overall health of patients by strengthening their stress adaptation capacity and intervening with depression.

## 1 Introduction

Driven by the rapid aging of the global population, multimorbidity (the coexistence of two or more chronic conditions) (1) has emerged as a preeminent challenge in geriatric health management, affecting over 51% of individuals aged 60 and older (2). Within this demographic, complex multimorbidity (CMM)—defined as the presence of four or more chronic diseases or the involvement of multiple organ systems (3, 4)—presents a distinctively intricate pathophysiology. Unlike general comorbidity, CMM imposes a substantial treatment burden (5). This burden manifests not only through frequent clinical visits and intricate polypharmacy regimens but also via the rigorous demands of lifestyle modifications and the cumulative economic and temporal costs associated with disease management (6).

Given the typically incurable nature of CMM, therapeutic goals have shifted from cure to fostering high-quality adaptation to “living with conditions” (7). This paradigm aligns fundamentally with Roy’s Adaptation Model (RAM), which conceptualizes health as the outcome of dynamic interactions between environmental stimuli and individual regulatory processes (8). Within this theoretical framework, treatment burden is distinctively conceptualized not merely as a stressor, but as the focal stimulus—the internal or external stimulus most immediately confronting the individual (9). Crucially, RAM posits that the impact of a focal stimulus on adaptive outcomes cannot directly manifest and must be transmitted sequentially through the internal coping control process, which comprises the regulator and cognator subsystems operating in tandem. Drawing on RAM’s outcome classification framework, effective regulation and regulatory failure represent two distinct manifestations of this coping control process. Applied to older adults with CMM, we propose that stress adaptation capacity represents effective regulation (10), wherein adequate cognitive-emotional resources enable patients to appraise and manage treatment demands without functional compromise. Conversely, depressive symptoms represent regulatory failure, signaling that the focal stimulus has overwhelmed internal coping capacities and disrupted the self-concept mode (11). Notably, RAM states that holistic adaptive health outcomes depend on the integrated functioning of the complete coping system, not any single regulatory pathway. Extending this premise, adaptation is jointly shaped by the relative balance of effective and failed regulation, rather than either pathway alone. RAM provides no theoretical presumption that positive and negative regulatory pathways exert equal predictive effects on overall adaptation; this unaddressed empirical question is tested exclusively among older adults with CMM in our study.

**Figure 1:**
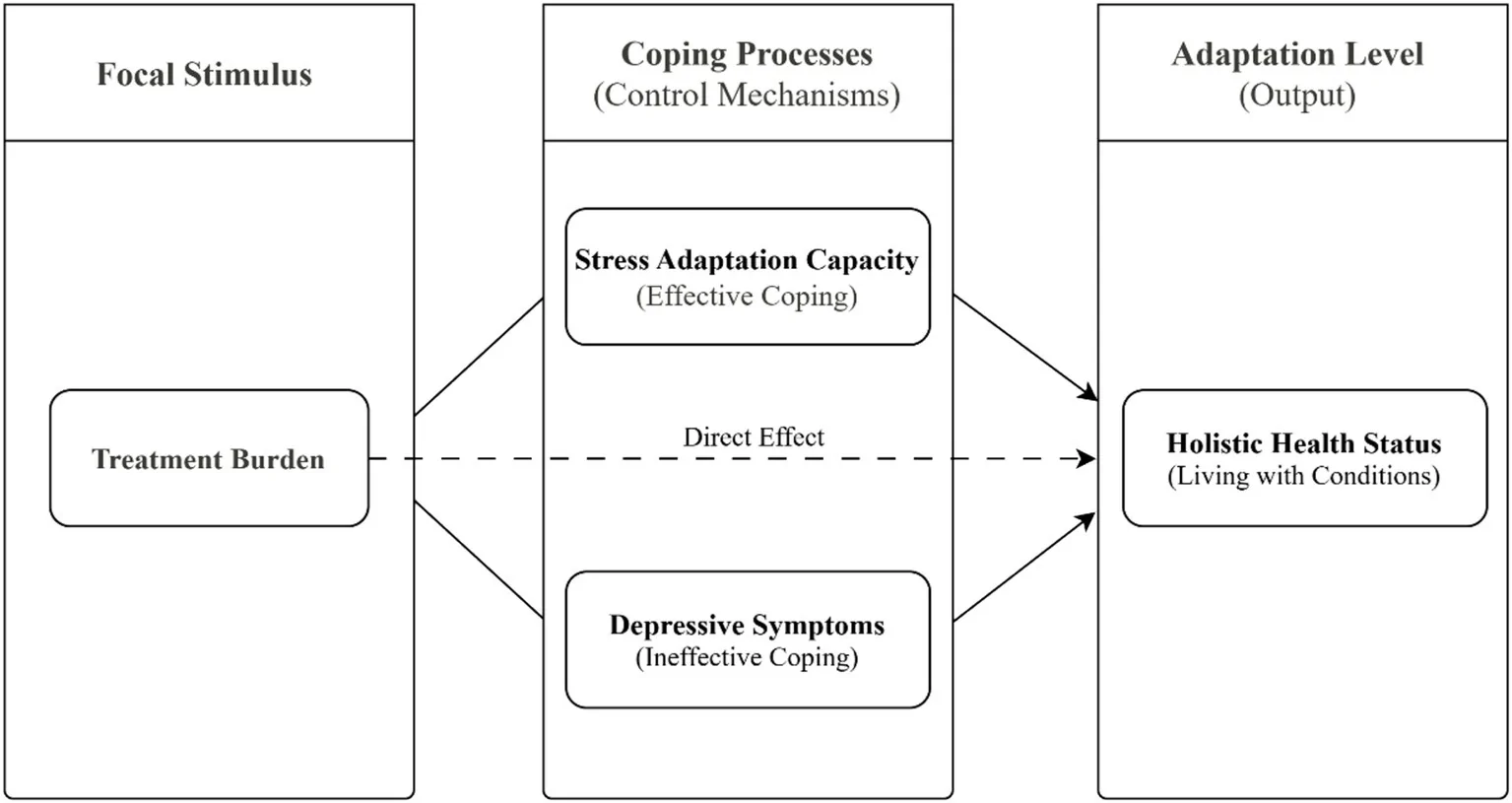
The conceptual framework (mapping of study variables onto the Roy Adaptation Model).

Even with this complete theoretical framework available, prior studies (12–14) have established bivariate associations among treatment burden, psychological distress, and health outcomes in chronic illness; however, the mechanistic pathway integrating these constructs remains systematically unverified. This gap is particularly evident within the vulnerable population of older adults with CMM. Crucially, no study has simultaneously modeled both regulatory pathways within a single analytical framework to test their relative contributions, nor have the shared unmeasured antecedents between stress adaptation and depression been adequately controlled. To address this, this study explicitly maps the empirical variables onto the theoretical constructs of RAM (Figure 1): treatment burden serves as the focal stimulus, while depressive symptoms and stress adaptation capacity function as parallel mediating regulatory responses that represent ineffective and effective regulation, respectively, and through which the stimulus exerts its effect on adaptation. By strictly testing the logical sequence from the focal stimulus to dual regulatory pathways and finally to adaptation outcomes, we quantify the relative predictive magnitude of each pathway to unpack the underlying mechanisms linking treatment burden and quality of life, and deliver targeted precision intervention references for multimorbid older adults.

## 2 Methods

### 2.1 Participants

The research team employed convenience sampling to recruit participants from nine healthcare facilities situated in three cities within the Zhejiang Province of China, from August 2022 to May 2023. The facilities included one provincial tertiary hospital, three county-level tertiary hospitals, three urban community healthcare centers, and two rural community healthcare centers. Eligible participants were both inpatients receiving clinical treatment and physical examination attendees who visited the above institutions during the survey period. Inclusion criteria were as follows: (1) age≥60 years; (2) Self-reported CMM (*n* ≥ 4 chronic diseases), with participants confirming that all chronic conditions had received formal clinical diagnoses from qualified physicians at regular medical institutions. During on-site screening, trained researchers briefly cross-checked participants’ outpatient medical records, discharge summaries, or diagnostic certificates they carried to exclude false self-reported disease information and ensure reporting reliability; and (3) willingness to participate in the study with signed informed consent. Exclusion criteria included: (1) communication barriers or a history of mental illness. This study was approved by the Ethics Committee of Zhejiang University School of Medicine [ID: 2022–006]. During the survey, the researchers explained the purpose and significance of the study to the participants, and all participants provided informed consent. Given that path analysis was used to explore the relationships among core variables, the sample size was determined based on the statistical requirements for structural equation modeling, which should be 10 to 20 times the number of estimated parameters (15). According to the sample size estimation principle of cross-sectional studies, the sample size should be 5 to 10 times the number of independent variables. This study included 11 independent variables. Considering a 20% sample loss rate, the sample size was calculated to be 69 to 138. Considering the need for the construction of the mediation model (16), the sample size should be at least 200 cases. A total of 507 patients were ultimately included in this study.

### 2.2 Measurements

#### 2.2.1 Socio-demographic data

A self-designed basic information questionnaire was used to collect socio-demographic information from the participants, including age, gender, ethnic, marital status, occupation, per-capita monthly household income, and educational level.

#### 2.2.2 Assessment of treatment burden

Treatment burden refers to the personal workload generated by patients in coping with and managing the treatment of multiple chronic diseases, as well as its impact on function and health (17). The Treatment Burden Questionnaire (TBQ) (18) was used to measure treatment burden. This instrument consists of 15 items across four dimensions: medication use, medical visits and follow-ups, medical-related and lifestyle changes, and health problems. Each item is scored on a scale from 0 to 10, with higher scores indicating greater burden (ranging from “no burden at all” to “extreme burden”). The total TBQ score is the sum of all item scores, ranging from 0 to 150, with higher total scores indicating a higher level of treatment burden. The scale demonstrated good internal consistency (Cronbach’s *α* = 0.890) and acceptable reliability of the retest (intraclass correlation coefficient = 0.760, 95% CI 0.670–0.830).

#### 2.2.3 Assessment of depressive symptoms

The 9-item Patient Health Questionnaire (PHQ-9) is a brief self-report tool measuring depressive symptoms experienced over the previous 2 weeks (19). Each item is rated on a 4-point Likert scale ranging from 0 (“not at all”) to 3 (“nearly every day”), generating a total score spanning 0–27. We adopted the internationally recognized severity stratification cutoffs originally proposed by Kroenke et al.: 0–4 for minimal depressive symptoms, 5–9 for mild, 10–14 for moderate, and ≥15 for severe depressive symptoms (19). The Chinese PHQ-9 version has demonstrated sound reliability and validity in domestic populations (20). In the present study, the PHQ-9 demonstrated excellent internal consistency (Cronbach’s *α* = 0.890).

#### 2.2.4 Assessment of stress adaptation capacity

The Stress Adaptation Scale (SAS) was developed by Smith et al. (10, 21) and comprises 12 items across two dimensions: resilience and thriving. The scale employs a 5-point Likert rating scale, with higher scores indicating better stress adaptation capacity. The psychometric properties of the original SAS demonstrated good reliability, with Cronbach’s *α* coefficients of 0.856 for the psychological resilience dimension, 0.858 for the psychological flourishing dimension, and 0.888 for the total scale. In this study, the Chinese version of the SAS (22) was utilized to assess stress adaptation capacity in patients with multimorbidity. The Chinese version of the SAS has been adapted to include 10 items, with a Cronbach’s *α* coefficient of 0.849 and a split-half reliability of 0.873. Content validity indices for all items and the total scale, as determined by expert ratings, exceeded 0.80, indicating satisfactory content validity.

#### 2.2.5 Assessment of holistic health status

The 20-item Long-Term Conditions Questionnaire (LTCQ) is a unidimensional measure that quantifies patients’ lived experience in multiple key areas—including treatment burden, dignity, support, coping, empowerment, knowledge, self-management, social participation, shame, family adaptation, and loneliness (23). We used the validated Chinese version (LTCQ-C) (24). Responses are given on a 5-point Likert scale and summed to a 0–100 score, with higher values indicating better adaptation to chronic disease. In the current study, the LTCQ-C demonstrated excellent internal consistency (Cronbach’s *α* = 0.926), good test–retest reliability (intraclass correlation coefficient = 0.829), and satisfactory split-half reliability (Spearman–Brown coefficient = 0.878).

### 2.3 Quality control

Investigators were trained uniformly before the survey to ensure standardization of the research process, thereby guaranteeing the consistency and comparability of the survey results. During the questionnaire survey, researchers checked the completeness of each item and promptly supplemented any missing information. A data quality monitoring mechanism was established. Data entry was performed using Epidata software to ensure data reliability. The entered data were strictly reviewed to check for outliers, logical inconsistencies, and other issues, and questionnaires with incomplete key information or answers showing obvious patterns were promptly corrected or removed.

### 2.4 Statistical analysis

Statistical analyses were performed in R 4.5.1. Continuous variables are expressed as mean ± SD or median [IQR] according to Shapiro–Wilk normality. Categorical variables are reported as *n* (%). Associations were examined with Pearson’s (normal) or Spearman’s (non-normal) correlation. Between-group comparisons of continuous outcomes used an independent-samples *t*-test or one-way ANOVA when normality was satisfied, and Mann–Whitney U or Kruskal–Wallis H test otherwise, to identify sociodemographic determinants of holistic health status in older adults with multimorbidity. Variables found to be statistically significant in the univariate analyses were subsequently included in multiple linear regression analyses for further investigation.

The parallel mediation model was constructed using the lavaan package (25). We first employed maximum likelihood estimation with robust standard errors (MLR) to accommodate potential non-normal distributions and missing data when evaluating overall model fit and direct/partial path coefficients. Before model estimation, all continuous predictors were standardized via z-score transformation, and categorical covariates were dummy-coded. To test the statistical significance of indirect mediating effects, we refitted the model using conventional maximum likelihood (ML) combined with 5,000 bias-corrected bootstrap resamples. To control for potential confounding effects, only sociodemographic variables that reached statistical significance in the multivariate linear regression model were incorporated into the final mediation model as adjustment covariates. Model goodness-of-fit was comprehensively assessed using standard indices, including the scaled *χ^2^/df* ratio, comparative fit index (CFI), root mean square error of approximation (RMSEA) with its 90% confidence interval, and standardized root mean square residual (SRMR). Bootstrap resampling with 5,000 bias-corrected iterations was performed to test the significance of indirect pathways, with a 95% confidence interval excluding zero indicating a significant mediating effect. A two-tailed *p*-value of less than 0.05 was considered statistically significant.

## 3 Result

### 3.1 General characteristics of the study population

A total of 550 questionnaires were distributed, and 507 valid responses were collected. Based on facility type and location, participants were classified into three institutional tiers: 132 from tertiary hospitals (comprising both provincial and county-level facilities), 315 from urban community healthcare centers, and 60 from rural/township community healthcare centers. Regarding age distribution, 65.1% of participants were aged 60–74 years, 30.2% were aged 75–84 years, and 4.7% were aged ≥85 years. The sample comprised 265 (52.3%) men and 242 (47.7%) women. The majority of participants (61.5%) reported a monthly household income of CNY 1,000–4,999, followed by 25.4% with an income of CNY 5,000–9,999. Educational attainment was generally low: 44.8% had completed primary school or less, and 48.3% had finished lower-secondary education. Further details are presented in Table 1.

**Table 1:** Descriptive statistics and univariate analysis of variables related to the holistic health status of the older adults with CMM (*n* = 507).

Variables	Category	*n*	LTCQ^a^	*Z/χ^2^*	*p*
Age	60–74	330 (65.1%)	79 (68, 89)	9.71	0.008
	75–84	153 (30.2%)	75 (62, 85)		
	≥85	24 (4.7%)	81 (67.25, 91.25)		
Sex	Male	265 (52.3%)	80 (69, 89)	−2.07	0.038
	Female	242 (47.7%)	76 (63, 88)		
Ethnicity	Han nationality	500 (98.6%)	78 (66, 88)	−0.54	0.591
	Others	7 (1.4%)	79 (75, 89)		
Marital status	Single	4 (0.8%)	63 (60.5, 71.25)	6.25	0.100
	Married	435 (85.8%)	79 (66, 88)		
	Divorced	4 (0.8%)	88 (79.75, 96.25)		
	Widowed	64 (12.6%)	75 (63.75, 82.5)		
Occupation	Government employees	4 (0.8%)	86.5 (74, 97.75)	74.46	<0.001
	Professional technicians	4 (0.8%)	85 (77.5, 92.25)		
	Retired personnel	314 (61.9%)	82 (71.25, 90)		
	Freelancers	22 (4.3%)	86.5 (66, 92)		
	Farmers	108 (21.3%)	68.5 (58, 76.25)		
	Industrial workers	8 (1.6%)	76.5 (66.75, 82)		
	Unemployed individuals	47 (9.3%)	68 (57.5, 81.5)		
Income	<1,000 CNY	50 (9.9%)	61 (51, 70.75)	63.82	<0.001
	1,000–4,999 CNY	312 (61.5%)	78.5 (67, 89)		
	5,000–9,999 CNY	129 (25.4%)	81 (71, 90)		
	≥10,000 CNY	16 (3.2%)	82 (72.5, 92.25)		
Education	Primary school or below	227 (44.8%)	75 (62, 87)	10.20	0.006
	Junior/high school and secondary vocational	245 (48.3%)	78 (69, 89)		
	College degree or higher	35 (6.9%)	81 (73.5, 90)		

a Median (interquartile range).

### 3.2 Correlation analysis of study variables

**Figure 2:**
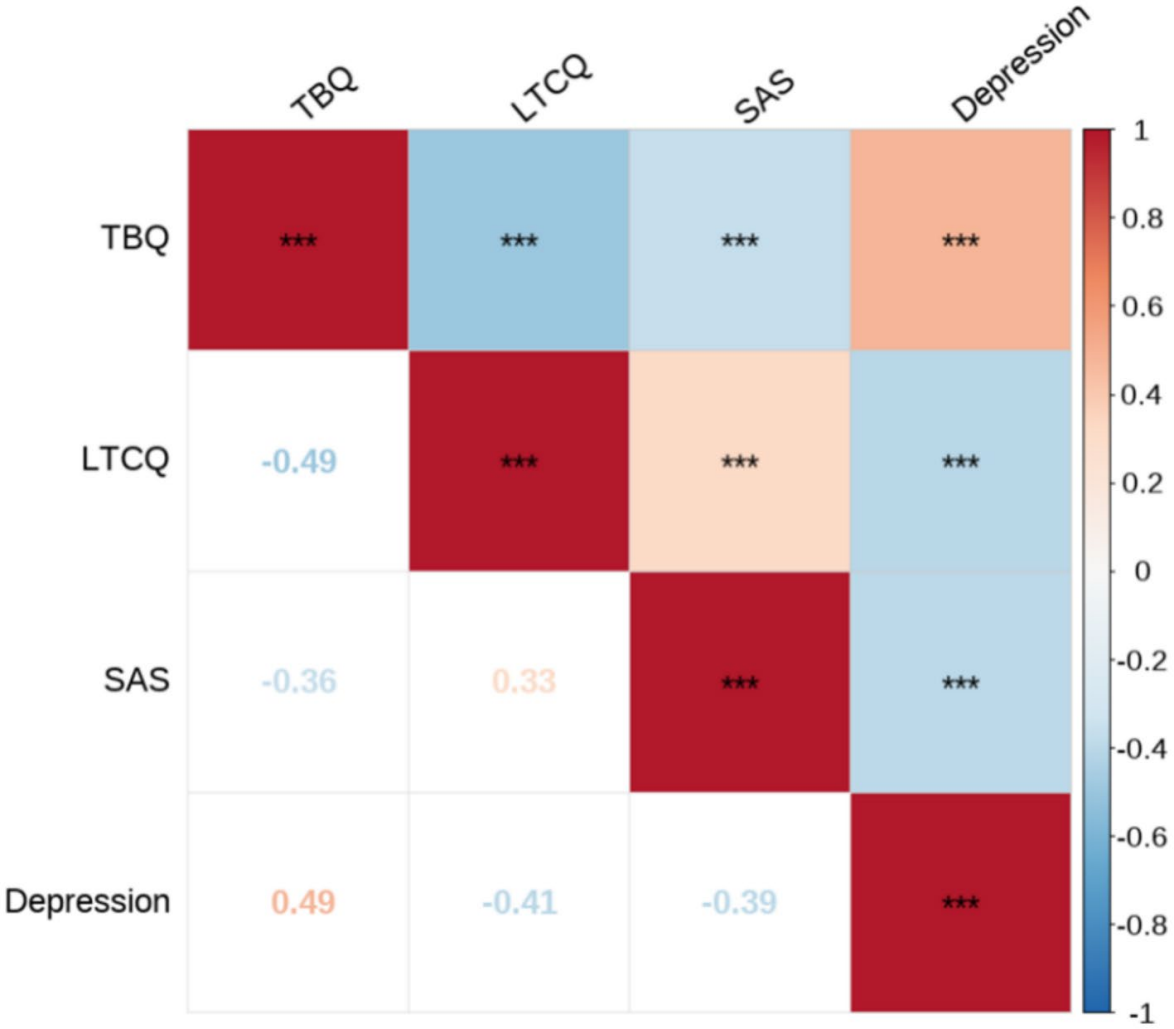
Correlation matrix of the four core variables (*n* = 507). ***, *p* < 0.001.

The participants exhibited the following scores for the core variables: treatment burden, 25.0 (IQR = 12.0–42.0); depressive symptoms, 3.0 (IQR = 1.0–6.0); stress adaptation capacity, 20.0 (IQR = 18.0–25.0); and holistic health status, 78.0 (IQR = 66.0–88.0). Correlation analysis revealed that treatment burden was negatively associated with both holistic health status (*r* = −0.49, *p* < 0.001) and stress adaptation capacity (*r* = −0.36, *p* < 0.001), whereas it was positively associated with depressive symptoms (*r* = 0.49, *p* < 0.001). Conversely, stress adaptation capacity was positively correlated with holistic health status (*r* = 0.33, *p* < 0.001). Depressive symptoms, however, showed inverse correlations with both stress adaptation capacity (*r* = −0.39, *p* < 0.001) and holistic health status (*r* = −0.41, *p* < 0.001). These bivariate associations are visualized in Figure 2.

### 3.3 Multivariate linear regression analysis

Table 2 presents the multiple linear regression results for holistic health status in older adults with CMM. The overall model was significant (*F* = 10.970, *p* < 0.001), and the generalized variance inflation factors were < 2 for all predictors, indicating the absence of serious multicollinearity. Compared with the youngest age group (60–74 years), patients aged 75–84 years reported significantly lower LTCQ scores (*B* = −3.492, *p* = 0.009). Similarly, female participants had lower scores than males (*B* = −3.577, *p* = 0.004). All higher-income categories were associated with progressively higher LTCQ scores (all *p* < 0.001 vs. the lowest-income group).

**Table 2:** Multiple linear regression analysis of variables related to holistic health status in older adults with CMM (*n* = 507).

Variables	*B*	SE	*t*	*p*	GVIF	*F*	Adjusted *R^2^*
Age					1.182	10.970***	0.216
75–84	−3.492	1.339	−2.607	0.009			
≥85	−1.517	2.997	−0.506	0.613			
Sex					1.115		
Female	−3.577	1.253	−2.855	0.004			
Occupation					1.878		
Government employees	6.398	9.663	0.662	0.508			
Professional technicians	−2.959	7.022	−0.421	0.674			
Retired personnel	−2.318	7.564	−0.306	0.759			
Freelancers	−9.662	7.271	−1.329	0.185			
Farmers	−6.343	8.511	−0.745	0.456			
Industrial workers	−12.749	7.327	−1.740	0.083			
Income					1.854		
1,000–4,999 CNY	14.019	2.364	5.930	<0.001			
5,000–9,999 CNY	16.547	2.707	6.113	<0.001			
≥10,000 CNY	19.369	4.133	4.686	<0.001			
Education					1.485		
Secondary education	−0.417	1.361	−0.306	0.759			
College degree or higher	0.620	2.716	0.228	0.819			

GVIF, generalized variance inflation factor; SE, Standard error; ***, *p* < 0.001.

### 3.4 Parallel mediation analysis of treatment burden and holistic health status in older adults

**Figure 3:**
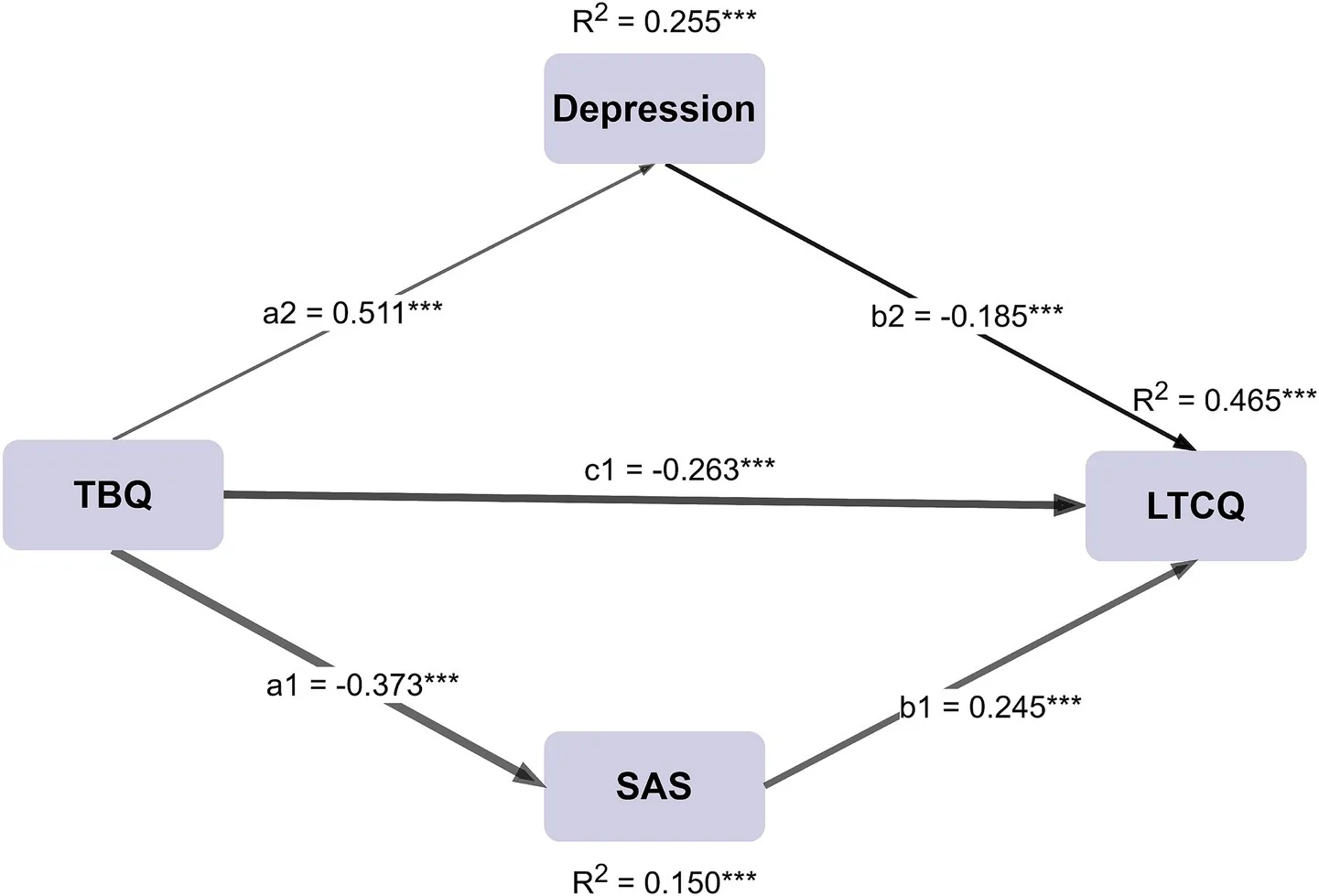
The parallel mediating effect model of treatment burden and holistic health status. *** *p* < 0.001.

The parallel mediation model exhibited favorable global fit: scaled *χ*^2^(5) = 5.72, *p* = 0.334, *χ^2^/df* = 1.14, CFI = 0.998, RMSEA = 0.020 (90% CI [0.000, 0.077]), SRMR = 0.025. Parallel mediation analysis revealed that after adjusting for sociodemographic confounders (sex, age group, and household income), treatment burden had significant indirect associations with holistic health status via two separate psychosocial pathways (Figure 3; Table 3). Specifically, treatment burden showed a significant direct association with holistic health status (c₁ = −0.263, 95% CI [−0.357, −0.168], *p* < 0.001). For the first indirect pathway, higher treatment burden was positively associated with depressive symptoms (a₂ = 0.511, 95% CI [0.410, 0.620], *p* < 0.001), which in turn was associated with poorer holistic health status (b₂ = −0.185, 95% CI [−0.271, −0.095], *p* < 0.001). The indirect effect through depressive symptoms was −0.095 (95% CI [−0.145, −0.047]), accounting for 21.2% of the total effect. For the second indirect pathway, treatment burden was negatively associated with stress adaptation (a₁ = −0.373, 95% CI [−0.472, −0.275], *p* < 0.001), whereas higher stress adaptation predicted better holistic health status (b₁ = 0.245, 95% CI [0.148, 0.339], *p* < 0.001). The indirect effect via stress adaptation was −0.091 (95% CI [−0.138, −0.051]), representing 20.3% of the total effect.

**Table 3:** Results of mediation analysis of holistic health status in the older adults with CMM.

Path relationship	*B*	SE	*t*	Bootstrap (95%CI)
TBQ → SAS	−0.373	0.05	−7.387***	[−0.472, −0.275]
TBQ → Depression	0.511	0.053	9.676***	[0.410, 0.620]
SAS → LTCQ	0.245	0.049	4.971***	[0.148, 0.339]
Depression → LTCQ	−0.185	0.045	−4.143***	[−0.271, −0.095]
TBQ → LTCQ (Direct)	−0.263	0.049	−5.392***	[−0.357, −0.168]
TBQ → SAS → LTCQ (Indirect)	−0.091	0.022	−4.129***	[−0.138, −0.051]
TBQ → Depression → LTCQ (Indirect)	−0.095	0.025	−3.769***	[−0.145, −0.047]
TBQ → LTCQ (Total)	−0.449	0.046	−9.741***	[−0.538, −0.360]

TBQ, Treatment Burden Questionnaire; SAS, Stress Adaptation Scale; LTCQ, Long-Term Conditions Questionnaire; CI, bias-corrected bootstrap confidence interval (5,000 resamples); SE, standard error. *** *p* < 0.001.

## 4 Discussion

### 4.1 Summary of key findings

Our findings confirm that treatment burden functions as a pervasive focal stimulus significantly associated with compromised holistic health in this vulnerable population. Crucially, parallel mediation analysis suggests that this negative association may be explained by two distinct internal coping processes: the presence of depressive symptoms (representing ineffective coping) and lower stress adaptation capacity (representing diminished effective coping resources). These findings provide empirical support for the “stimulus-coping-adaptation” trajectory as a key framework for understanding quality of life in CMM.

### 4.2 The direct association: treatment burden as an overwhelming focal stimulus

Our results demonstrated a significant direct negative association between treatment burden and holistic health status, aligning with previous research highlighting the “workload-capacity” imbalance in chronic care (26). This cross-sectional correlation links to heavy “patient work” among older adults with multimorbidity—addressing the fragmentation of the medical service system, managing polypharmacy, and adhering to strict lifestyle interventions, which together form a major therapeutic burden (27, 28). Prior studies confirm that intensive regimens impose financial, time, and cognitive strains, cross-sectionally correlated with less participation in valued daily activities (29). Theoretically, participants with workload exceeding personal adaptive thresholds show reduced social and meaningful life engagement alongside poorer holistic health. These concurrent patterns suggest intensive treatment regimens act as key stressors co-occurring with compromised overall health under high treatment burden (30).

### 4.3 The destructive pathway: the mediating role of depressive symptoms

We identified a significant indirect pathway wherein treatment burden is associated with higher levels of depressive symptoms, which are concurrently linked to poorer holistic health. This relationship may be understood through two key mechanisms. First, from a psychological perspective, the unremitting demands of complex regimens can engender a profound sense of “powerlessness” and “loss of control” (31). When patients feel that their daily lives are dominated by medical tasks despite their best efforts, this perceived lack of control may serve as a potential contributor to depressive symptoms (32). Second, from a behavioral perspective, the sheer volume of “patient work” (e.g., frequent visits, medication management) inevitably competes with other life roles. The time and energy consumed by treatment tasks often coincide with reduced social interactions and leisure activities, leading to social isolation (33). This loss of social support and meaningful engagement may further correlate with depressive states. Ultimately, these depressive symptoms may erode the motivation and executive function required for self-management, creating a maladaptive cycle: high burden co-occurs with depression, potentially involving feelings of powerlessness as an underlying mechanism and isolation, while depression is simultaneously associated with greater difficulty in “living well” with chronic conditions.

### 4.4 The depletion pathway: the mediating role of stress adaptation capacity

This study identifies stress adaptation capacity as a second significant mediator, revealing a critical depletion pathway. Stress adaptation capacity is fundamentally a protective psychological resource that helps patients buffer against life stressors. However, in the context of excessive treatment burden, this protective mechanism may not function effectively: higher levels of treatment burden tend to co-occur with diminished internal cognitive coping reserves. When such reserves are at lower levels, patients’ flexibility in responding to health challenges is correspondingly reduced, which is in turn linked to poorer adaptation outcomes (34). Notably, the proportion of the mediation effect via the stress adaptation pathway (20.3%) closely approximates that of the depressive symptom pathway (21.2%), indicating that deficits in positive psychological resources and negative depressive emotions exhibit comparable associations with overall health. This finding suggests that while depressive symptoms serve as overt pathological signals readily captured by routine screening tools, the depletion of adaptive reserve represents a latent functional deficit often obscured by an apparent absence of depression (35). If clinical care focuses exclusively on alleviating depressive symptoms, it may not only fail to adequately mitigate health impairments mediated by psychosocial factors but also overlook high-risk individuals whose emotional presentation appears stable despite critically depleted internal reserves. Consequently, the management of older adults with multimorbidity must shift from isolated symptom control toward holistic support aimed at restoring psychological homeostasis. Specifically, alongside addressing depression, clinicians should proactively replenish patients’ adaptive reserves through strategies such as simplifying treatment regimens (36), empowering self-management, and strengthening social support networks. Such an approach fosters the intrinsic capacity needed to sustainably cope with treatment burden, rather than relying solely on external interventions to maintain transient stability.

### 4.5 Sociodemographic determinants of adaptation

Our analysis also revealed that advanced age (75–84 years), female gender, and lower income were associated with poorer holistic health. The disparity observed in low-income participants may reflect the “financial toxicity” of treatment burden, where economic constraints coincide with limited access to supportive resources, potentially amplifying the negative association between the focal stimulus and health outcomes (37). Similarly, older women may face a double burden of disease management and domestic caregiving roles, leaving them with fewer cognitive and physical resources for their own health adaptation (38). These findings suggest that CMM patients with these demographic profiles represent a high-risk subgroup requiring targeted support.

### 4.6 Clinical implications

The findings have significant implications for nursing practice and geriatric care. First, to address the focal stimulus, clinicians should consider embracing the principles of Minimally Disruptive Medicine (39). This involves routinely assessing treatment burden and prioritizing “deprescribing” or simplifying regimens to align with the patient’s capacity. Second, interventions could adopt a “dual-track” approach targeting coping processes: screening for and addressing depressive symptoms (targeting the destructive link) while simultaneously fostering stress adaptation capacity through resilience training or empowerment-based education (strengthening the protective link). Finally, nurses should pay special attention to socially vulnerable groups (e.g., low-income, oldest-old) to ensure equity in health adaptation outcomes. Despite its strengths, this study has several limitations. First, selection bias may arise from convenience sampling across three cities within a single province, limiting generalizability and potentially leading to an underestimation of the true association between treatment burden and holistic health status. Second, although we adjusted for age and gender, residual confounding cannot be excluded; unmeasured factors such as objective disease severity and caregiver support may simultaneously influence both psychosocial mediators and health outcomes. Third, while medical records were reviewed to verify self-reported diagnoses, reliance on self-report for other variables may still introduce common method and recall bias, affecting the accuracy. Finally, the cross-sectional design precludes causal inference regarding the identified psychosocial pathways. Future studies should employ longitudinal, multi-center designs with objective measures to validate these findings in diverse populations.

## 5 Conclusion

This study examined the relationship between treatment burden and holistic health status in older adults with CMM and analyzed the mediating roles of depressive symptoms and stress adaptation capacity. The results demonstrated a direct negative association between treatment burden and holistic health status, with depressive symptoms and stress adaptation capacity serving as partial mediators. Therefore, in clinical practice, early identification and intervention for depressive symptoms should be emphasized, and higher stress adaptation capacity may be associated with better holistic health status among patients experiencing high treatment burden.

## Data Availability

The raw data supporting the conclusions of this article will be made available by the authors, without undue reservation.

## References

[1] Skou ST Mair FS Fortin M Guthrie B Nunes BP Miranda JJ . Multimorbidity. Nat Rev Dis Primers. (2022) 8:48. doi: 10.1038/s41572-022-00376-4 35835758 PMC7613517

[2] Chowdhury SR Chandra Das D Sunna TC Beyene J Hossain A. Global and regional prevalence of multimorbidity in the adult population in community settings: a systematic review and meta-analysis. EClinicalMedicine. (2023) 57:101860. doi: 10.1016/j.eclinm.2023.101860, 36864977 PMC9971315

[3] Kivimäki M Strandberg T Pentti J Nyberg ST Frank P Jokela M . Body-mass index and risk of obesity-related complex multimorbidity: an observational multicohort study. Lancet Diabetes Endocrinol. (2022) 10:253–63. doi: 10.1016/S2213-8587(22)00033-X, 35248171 PMC8938400

[4] The Academy of Medical Sciences. Multimorbidity: a Priority for Global Health Research. London: Academy of Medical Sciences (2018).

[5] Makovski TT Schmitz S Zeegers MP Stranges S Van Den Akker M. Multimorbidity and quality of life: systematic literature review and meta-analysis. Ageing Res Rev. (2019) 53:100903. doi: 10.1016/j.arr.2019.04.005, 31048032

[6] Vetrano DL Rizzuto D Calderón-Larrañaga A Onder G Welmer A-K Bernabei R . Trajectories of functional decline in older adults with neuropsychiatric and cardiovascular multimorbidity: a Swedish cohort study. PLoS Med. (2018) 15:e1002503. doi: 10.1371/journal.pmed.1002503, 29509768 PMC5839531

[7] Li L Chen D Zhou M Zhang H Zheng R Su J . Global research trends in chronic care for comorbid allergic diseases: a web of science bibliometric review (1999–2024). Curr Allergy Asthma Rep. (2025) 25:58. doi: 10.1007/s11882-025-01238-1, 41361064

[8] Wang X Ye Z. Development of a middle-range theory of adaptation to chronic illness based on the Roy’s model. Chin J Nurs. (2021) 56:1193–200. doi: 10.3761/j.issn.0254-1769.2021.08.012

[9] Rogers EA Abi H Linzer M Eton DT. Treatment burden in people with hypertension is correlated with patient experience with self-management. J Am Board Fam Med. (2021) 34:1243–5. doi: 10.3122/jabfm.2021.06.210191, 34772780 PMC9110114

[10] Smith BW Dalen J Wiggins K Tooley E Christopher P Bernard J. The brief resilience scale: assessing the ability to bounce back. Int J Behav Med. (2008) 15:194–200. doi: 10.1080/10705500802222972, 18696313

[11] Yang C Zhu S Hui Z Mo Y. Psychosocial factors associated with medication burden among community-dwelling older people with multimorbidity. BMC Geriatr. (2023) 23:741. doi: 10.1186/s12877-023-04444-6, 37964196 PMC10648314

[12] Gebreyohannes EA Gebresillassie BM Mulugeta F Dessu E Abebe TB. Treatment burden and health-related quality of life of patients with multimorbidity: a cross-sectional study. Qual Life Res. (2023) 32:3269–77. doi: 10.1007/s11136-023-03473-3, 37405663 PMC10522511

[13] Van Wilder L Devleesschauwer B Clays E Pype P Vandepitte S De Smedt D. Polypharmacy and health-related quality of life/psychological distress among patients with chronic disease. Prev Chronic Dis. (2022) 19:220062. doi: 10.5888/pcd19.220062, 35980834 PMC9390791

[14] Falke C Karapinar F Bouvy M Emmelot M Belitser S Boland B . The association between medication use and health-related quality of life in multimorbid older patients with polypharmacy. Eur Geriatr Med. (2024) 15:1713–23. doi: 10.1007/s41999-024-01036-4, 39162972 PMC11631809

[15] Jackson DL. Revisiting sample size and number of parameter estimates: some support for the N:q hypothesis. Struct Equ Model Multidiscip J. (2003) 10:128–41. doi: 10.1207/S15328007SEM1001_6

[16] Hoyle RH Gottfredson NC. Sample size considerations in prevention research applications of multilevel modeling and structural equation modeling. Prev Sci. (2015) 16:987–96. doi: 10.1007/s11121-014-0489-8, 24752569 PMC4207737

[17] Eton D Ramalho De Oliveira D Egginton J Ridgeway J Odell L May C . Building a measurement framework of burden of treatment in complex patients with chronic conditions: a qualitative study. Patient Relat Outcome Meas. (2012) 3:39. doi: 10.2147/PROM.S34681, 23185121 PMC3506008

[18] Tran V-T Montori VM Eton DT Baruch D Falissard B Ravaud P. Development and description of measurement properties of an instrument to assess treatment burden among patients with multiple chronic conditions. BMC Med. (2012) 10:68. doi: 10.1186/1741-7015-10-68, 22762722 PMC3402984

[19] Kroenke K Spitzer RL Williams JBW. The PHQ-9: validity of a brief depression severity measure. J Gen Intern Med. (2001) 16:606–13. doi: 10.1046/j.1525-1497.2001.016009606.x, 11556941 PMC1495268

[20] Xiong N Fritzsche K Wei J Hong X Leonhart R Zhao X . Validation of patient health questionnaire (PHQ) for major depression in Chinese outpatients with multiple somatic symptoms: a multicenter cross-sectional study. J Affect Disord. (2015) 174:636–43. doi: 10.1016/j.jad.2014.12.042, 25576931

[21] Smith BW Decruz-Dixon N Schodt K Schodt K Torres F. Handbook of Assessment in Mindfulness Research. Cham: Springer International Publishing (2023).

[22] Fu Y Wu J Zhao B Lai C Xue E Wang D . Development of a Chinese version of the stress adaption scale and the assessment of its reliability and validity among Chinese patients with multimorbidity. J Zhejiang Univ (Med Sci). (2023) 52:361–70. doi: 10.3724/zdxbyxb-2022-0721, 37476947 PMC10409896

[23] Peters M Potter C Kelly L Hunter C Jenkinson C Coulter A . The long-term conditions questionnaire: conceptual framework and item development. Patient Relat Outcome Meas. (2016) 7:109–25. doi: 10.2147/PROM.S104552, 27621678 PMC5012841

[24] Lai C Ye Z Shao J Wu J Zhao B Fu Y . Development and testing of the reliability and validity of a Chinese version of the long-term conditions questionnaire. J Zhejiang Univ (Med Sci). (2023) 52:371–8. doi: 10.3724/zdxbyxb-2023-0029, 37476948 PMC10409922

[25] Rosseel Y. Lavaan: an R package for structural equation modeling. J Stat Softw. (2012) 48:1–36. doi: 10.18637/jss.v048.i02

[26] Morris JE Roderick PJ Harris S Yao G Crowe S Phillips D . Treatment burden for patients with multimorbidity: cross-sectional study with exploration of a single-item measure. Br J Gen Pract. (2021) 71:e381–90. doi: 10.3399/BJGP.2020.0883, 33875419 PMC8074644

[27] Gobeil-Lavoie AP Chouinard MC Danish A Hudon C. Characteristics of self-management among patients with complex health needs: a thematic analysis review. BMJ Open. (2019) 9:e028344. doi: 10.1136/bmjopen-2018-028344, 31129599 PMC6538095

[28] Tran V-T Montori VM Ravaud P. Is my patient overwhelmed?: determining thresholds for acceptable burden of treatment using data from the ComPaRe e-cohort. Mayo Clin Proc. (2020) 95:504–12. doi: 10.1016/j.mayocp.2019.09.004 31619365

[29] Dhillon H Nordin RB Ramadas A. Quality of life and associated factors among primary care Asian patients with type 2 diabetes mellitus. Int J Environ Res Public Health. (2019) 16:3561. doi: 10.3390/ijerph16193561, 31547629 PMC6801549

[30] Lee JE Lee J Shin R Oh O Lee KS. Treatment burden in multimorbidity: an integrative review. BMC Prim Care. (2024) 25:352. doi: 10.1186/s12875-024-02586-z, 39342121 PMC11438421

[31] Zheng Y Lin P Zhao L Qian T Xie J Xu L. Multimorbidity is significantly associated with higher prevalence of depressive symptoms in middle-aged and older Chinese adults. Prev Med Rep. (2025) 60:103289. doi: 10.1016/j.pmedr.2025.103289, 41278617 PMC12634280

[32] Read JR Sharpe L Modini M Dear BF. Multimorbidity and depression: a systematic review and meta-analysis. J Affect Disord. (2017) 221:36–46. doi: 10.1016/j.jad.2017.06.009, 28628766

[33] Hounkpatin H Simpson G Santer M Farmer A Dambha-Miller H. Multiple long-term conditions, loneliness and social isolation: a scoping review of recent quantitative studies. Arch Gerontol Geriatr. (2024) 120:105347. doi: 10.1016/j.archger.2024.105347, 38309103

[34] Guo J Yang J Wiley J Ou X Zhou Z Whittemore R. Perceived stress and self-efficacy are associated with diabetes self-management among adolescents with type 1 diabetes: a moderated mediation analysis. J Adv Nurs. (2019) 75:3544–53. doi: 10.1111/jan.14179, 31441523

[35] Folorunsho S Lawal OP Ani NC Somuah MA. Functional impairment without depression diagnosis as an indicator of psychological distress in older adults. Glob Ment Health (Camb). (2025) 12:e138. doi: 10.1017/gmh.2025.10098, 41438275 PMC12720376

[36] Leppin A Montori V Gionfriddo M. Minimally disruptive medicine: a pragmatically comprehensive model for delivering care to patients with multiple chronic conditions. Healthcare. (2015) 3:50–63. doi: 10.3390/healthcare3010050, 27417747 PMC4934523

[37] Ni Y Zhou Y Kivimäki M Cai Y Carrillo-Larco RM Xu X . Socioeconomic inequalities in physical, psychological, and cognitive multimorbidity in middle-aged and older adults in 33 countries: a cross-sectional study. Lancet Healthy Longev. (2023) 4:e618–28. doi: 10.1016/S2666-7568(23)00195-2, 37924843

[38] Yao S-S Cao G-Y Han L Chen Z-S Huang Z-T Gong P . Prevalence and patterns of multimorbidity in a nationally representative sample of older Chinese: results from the China health and retirement longitudinal study. J Gerontol A Biol Sci Med Sci. (2020) 75:1974–80. doi: 10.1093/gerona/glz185 31406983

[39] Trevena L. Minimally disruptive medicine for patients with complex multimorbidity. Aust J Gen Pract. (2018) 47:175–9. doi: 10.31128/AFP-10-17-4374, 29621856

